# Aspirin therapy for cancer: it is never too late

**DOI:** 10.1038/bjc.2011.373

**Published:** 2011-10-11

**Authors:** J A Z Jankowski, P J Limburg

**Affiliations:** 1 Department of Oncology, Old Road Campus, Oxford OX3 7DQ, UK; 2 Division of Gastroenterology & Hepatology, Mayo Clinic, Rochester, MN, USA

Willow tree extracts have been recognised for their medicinal properties since 3500 BC (Mahdi *et al*, 2006). In the mid-late 1800s, a series of breakthrough investigations led to the isolation of salicin, synthesis of acetylsalicylic acid, and patent of aspirin. However, more than 100 years and 20 000+ scientific publications later, aspirin remains an interesting enigma, in that, the scope of biological activity and spectrum of clinical application for this agent remains incompletely defined. Aspirin's anti-carcinogenic potential was originally described in rodent models, with the Melbourne Colorectal Cancer Study credited with reporting the first human observational data in 1988 (Kune *et al*, 1988). Since then, the use of aspirin has been inversely associated with multiple target organ malignancies (Cuzick *et al*, 2009). However, existing data are not entirely consistent, with unresolved questions regarding the dose, duration and follow-up period needed to fully appreciate aspirin's actual chemopreventive benefits. To address these questions, Rothwell *et al* (2011) recently combined and analysed data from eight randomised clinical trials of aspirin (dose range 75–1200 mg per day) *vs* placebo, conducted in patient populations at increased risk for cardiovascular disease, wherein the scheduled duration of intervention was at least 4 years. Salient findings from the pooled analysis included a 21% reduction in overall risk for death due to cancer (hazard ratio (HR)=0.79, 95% confidence interval (CI)=0.68–0.92; *P*=0.003) among subjects assigned to the active *vs* placebo interventions. Notably, aspirin therapy for at least 5 years resulted in even greater risk reduction (30–40%), with persistent effects on cancer mortality extending out to 20 years of follow-up (HR=0.80; 95% CI=0.72–0.88; *P*<0.0001 for solid organ tumours). Stratification by dose did not appreciably influence the observed risk estimates. In response to this intriguing report, discussions regarding the routine aspirin use for the primary purpose of favourably affecting cancer risk have been re-invigorated. Aside some benefits in cancer mortality seem to be apparent within 4 years, suggesting provocatively that the humble aspirin could perhaps also stifle cancer growth (Rothwell *et al*, 2011). However, despite the ever-mounting efficacy data, residual concerns remain about the potential of aspirin-associated adverse events. This issue continues to impede recommendations for widespread cancer chemoprevention in generally healthy adults (Jankowski and Hawk, 2006; Jankowski and Hunt, 2008; Rothwell *et al*, 2011). Specifically as about 30% of the population may get cancer, there is a need for prevention (Jankowski and Hawk, 2006). However, from estimates by Rothwell *et al* (2011), only 20% of the latter group will derive a chemopreventive benefit from aspirin. Furthermore, the serious risks of aspirin, usually GI bleeding, are currently contentious, ranging from 0.02 to 2% per year (Jankowski and Hawk, 2006; Jankowski and Hunt, 2008; Rothwell *et al*, 2011). This could mean that serious side effects from 10 years of aspirin chemoprevention would range from 0.2 to 20%. At the higher end, the benefits of chemoprevention would be nullified by the risks. The recent report from Langley *et al* (2011) is potentially paradigm-shifting, as it alludes to an improved risk benefit ratio for adjuvant aspirin therapy for early cancers This area has to date received relatively lesser attention compared with its chemopreventive potential. As discussed by the authors, new insights into the mechanisms of aspirin-mediated growth inhibition (such as altered endothelial cell tubule formation resulting in decreased angiogenesis) may afford unique advantages over other non-steroidal anti-inflammatory drugs or selective COX-2 inhibitors for treating malignant tumours. Other putative effects of COX-2 on apoptosis, invasion, and immunoregulation (Ghosh *et al*, 2010) support targeting this enzyme in advanced, adjuvant, or neoadjuvant chemotherapy settings as well. Available observational data further suggest that aspirin use *vs* non-use is associated with improved survival following colorectal (Chan *et al* 2009) or breast cancer (Holmes *et al* 2010) diagnosis. However, three small randomised, controlled trials of aspirin (dose range 1000–2400 mg per day) in combination with other anti-cancer therapies did not confirm the anticipated survival benefit. Similarly, results from the recently reported VICTOR Trial (Midgley *et al* 2010), which evaluated the effects of rofecoxib (a selective COX-2 inhibitor) 20 mg per day *vs* placebo on overall survival among stages II and III colorectal cancer patients who had undergone potentially curative surgery and completed adjuvant chemotherapy (when indicated), also failed to demonstrate a statistically significant advantage from the active agent, although the intervention phase was terminated early (median exposure time of 7.4 months) due to concerns regarding increased cardiovascular toxicity.

Given the current evidence base, where should additional resources be invested to decipher aspirin's true anti-cancer potential? As noted by Langley *et al* (2011), further investigation of the benefits and harms associated with aspirin therapy in high-risk subject cohorts (i.e., patients with newly diagnosed cancer or established premalignant conditions) may permit re-calibration of an ‘acceptable’ safety profile. Consideration of oncologic and non-oncologic endpoints, relating to efficacy and toxicity, in all large clinical trials also seems imperative. Ideally, these disparate endpoints could be accurately measured and appropriately weighted to more appropriately summarise the aspirin's total impact on personal and/or public health. Specific to the adjuvant therapy setting, broader use of molecular phenotyping to define tumours that are likely to be most susceptible to aspirin exposure (i.e., based on increased COX-2 expression) could improve the response rate. Finally, further attempts to clarify the genetic characteristics that differentiate aspirin responders from non-responders may also be of merit. Several of these issues will be directly assessed by ongoing prospective studies, such as the AspECT aspirin chemoprevention trial as well as the ChOPIN genetic trial (Moayyedi and Jankowski, 2010). It may even be possible to predict who will get a response to aspirin, based upon their genome.

In summary, the case for aspirin therapy continues to get stronger. It may never be too late for aspirin adjuvant therapy, even if chemoprevention is not feasible. However, more well-designed randomised controlled trials are needed with adequate follow-up time before definitive clinical recommendations can be given.
